# (*E*)-6-Bromo-3-{2-[2-(2-meth­oxy­benzyl­idene)hydrazin­yl]-1,3-thia­zol-4-yl}-2*H*-chromen-2-one

**DOI:** 10.1107/S1600536811024536

**Published:** 2011-06-25

**Authors:** Afsheen Arshad, Hasnah Osman, Chan Kit Lam, Madhukar Hemamalini, Hoong-Kun Fun

**Affiliations:** a School of Chemical Sciences, Universiti Sains Malaysia, 11800 USM, Penang, Malaysia; b School of Pharmaceutical Sciences, Universiti Sains Malaysia, 11800 USM, Penang, Malaysia; c X-ray Crystallography Unit, School of Physics, Universiti Sains Malaysia, 11800 USM, Penang, Malaysia

## Abstract

In the title compound, C_20_H_14_BrN_3_O_3_S, the mol­ecule adopts an *E* configuration about the central C=N double bond. The chromene ring system and the thia­zole ring are approximately planar [maximum deviations = 0.029 (3) and 0.007 (3) Å, respectively]. The chromene ring system is inclined at angles of 7.37 (12) and 13.90 (13)° with respect to the thia­zole and benzene rings, respectively, while the thia­zole ring makes a dihedral angle of 12.58 (15)° with the benzene ring. In the crystal, mol­ecules are connected by N—H⋯O hydrogen bonds, forming *C*(8) supra­molecular chains along the *c* axis.

## Related literature

For related structures, further synthetic details and background references, see: Arshad *et al.* (2011**a* ▶,b* ▶).
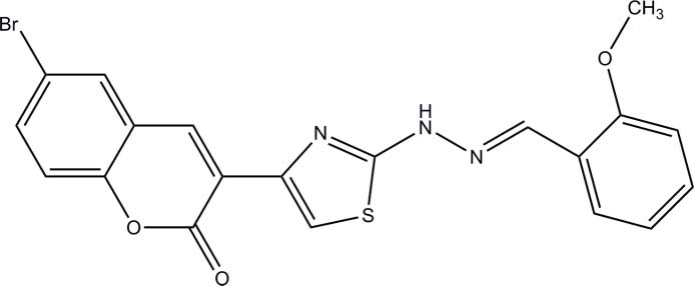


## Experimental

### 

#### Crystal data

C_20_H_14_BrN_3_O_3_S
*M* *_r_* = 456.31
Monoclinic, 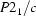

*a* = 7.2802 (12) Å
*b* = 19.551 (3) Å
*c* = 14.0638 (18) Å
β = 113.352 (7)°
*V* = 1837.8 (5) Å^3^
*Z* = 4
Mo *K*α radiation
μ = 2.38 mm^−1^
*T* = 296 K
0.43 × 0.07 × 0.04 mm

#### Data collection

Bruker APEXII DUO CCD diffractometer
Absorption correction: multi-scan (*SADABS*; Bruker, 2009 ▶) *T*_min_ = 0.427, *T*_max_ = 0.921
11951 measured reflections
4266 independent reflections
2786 reflections with *I* > 2σ(*I*)
*R*_int_ = 0.036

#### Refinement

*R*[*F* ^2^ > 2σ(*F* ^2^)] = 0.038
*wR*(*F* ^2^) = 0.089
*S* = 1.01
4266 reflections
253 parameters
H-atom parameters constrained
Δρ_max_ = 0.30 e Å^−3^
Δρ_min_ = −0.29 e Å^−3^

### 

Data collection: *APEX2* (Bruker, 2009 ▶); cell refinement: *SAINT* (Bruker, 2009 ▶); data reduction: *SAINT*; program(s) used to solve structure: *SHELXTL* (Sheldrick, 2008 ▶); program(s) used to refine structure: *SHELXTL*; molecular graphics: *SHELXTL*; software used to prepare material for publication: *SHELXTL* and *PLATON* (Spek, 2009 ▶).

## Supplementary Material

Crystal structure: contains datablock(s) global, I. DOI: 10.1107/S1600536811024536/hb5923sup1.cif

Structure factors: contains datablock(s) I. DOI: 10.1107/S1600536811024536/hb5923Isup2.hkl

Supplementary material file. DOI: 10.1107/S1600536811024536/hb5923Isup3.cml

Additional supplementary materials:  crystallographic information; 3D view; checkCIF report

## Figures and Tables

**Table 1:** Hydrogen-bond geometry (Å, °)

*D*—H⋯*A*	*D*—H	H⋯*A*	*D*⋯*A*	*D*—H⋯*A*
N2—H1⋯O2^i^	0.94	2.10	3.021 (3)	164

Symmetry code: (i) 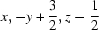.

**Fig. 1.**
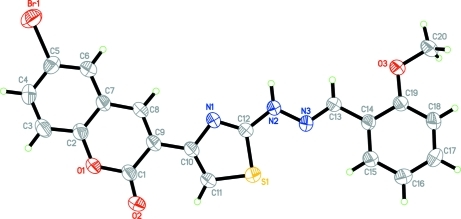
The asymmetric unit of the title compound, showing 50% probability displacement ellipsoids and the atom-numbering scheme.

**Fig. 2.**
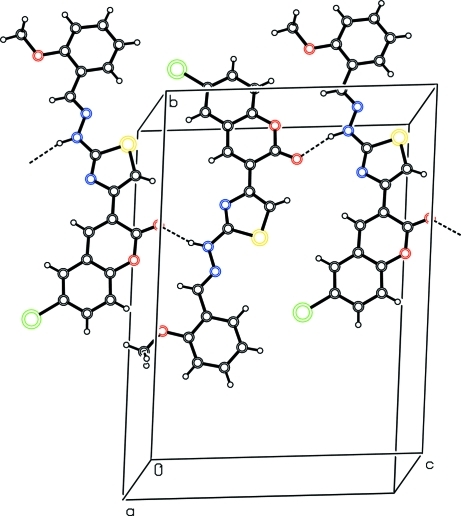
A view of a one-dimensional supramolecular chain along the c-axis.

## supplementary crystallographic information

### Comment

As part of our ongoing studies of substituted coumarins (Arshad *et al.*, 2011**a*,b*) we now present the crystal structure of the title compound, (I).

In (I), the molecule adopts an *E* configuration about the central C13═N3 double bond. The chromene (O1/C1–C9) and the thiazole (S1/N1/C10–C12) rings are  approximately planar [maximum deviations of  0.029 (3) Å  for atom C4 and 0.007 (3) Å for atom C12, respectively]. The chromene (O1/C1–C9) ring system is inclined at angles of 7.37 (12)° and 13.90 (13)° with respect to the thiazole (S1/N1/C10–C12) and benzene (C14–C19) rings, respectively, while the thiazole (S1/N1/C10–C12) ring makes a dihedral angle of 12.58 (15)° with the benzene ((C14–C19) ring.

In the crystal (Fig. 2), the molecules are connected by N2—H1···O2 (Table 1) hydrogen bonds forming supramolecular chains along the *c*-axis.

### Experimental

The title compound was synthesized by the same procedure as mentioned in our previous papers (Arshad *et al.*, 2011**a*,b*). 2-Methoxy benzylidene thiosemicarbazone was reacted with 6-bromo-3- (2-bromoacetyl)-2*H*-chromen-2-one in chloroform-ethanol (2:1) mixture. The reaction mixture was refluxed for 2–3 hours at 60°C to get dense yellow precipitates. It was cooled in ice bath and basified with ammonia to pH 7–8. The title compound (I) was recrystallized from CHCl_3_–EtOH (1:1) as golden yellow needles.

### Refinement

All hydrogen atoms were positioned geometrically [N–H = 0.9449 Å and C–H = 0.93 or 0.96 Å] and were refined using a riding model, with *U*_iso_(H) = 1.2 or 1.5 *U*_eq_(C). A rotating group model was applied to the methyl groups

### Crystal data

**Table:**

C_20_H_14_BrN_3_O_3_S	*F*(000) = 920
*M**_r_* = 456.31	*D*_x_ = 1.649 Mg m^−^^3^
Monoclinic, *P*2_1_/*c*	Mo *K*α radiation, λ = 0.71073 Å
Hall symbol: -P 2ybc	Cell parameters from 2197 reflections
*a* = 7.2802 (12) Å	θ = 3.0–22.4°
*b* = 19.551 (3) Å	µ = 2.38 mm^−^^1^
*c* = 14.0638 (18) Å	*T* = 296 K
β = 113.352 (7)°	Needle, yellow
*V* = 1837.8 (5) Å^3^	0.43 × 0.07 × 0.04 mm
*Z* = 4	

### Data collection

**Table:**

Bruker APEXII DUO CCD diffractometer	4266 independent reflections
Radiation source: fine-focus sealed tube	2786 reflections with *I* > 2σ(*I*)
graphite	*R*_int_ = 0.036
φ and ω scans	θ_max_ = 27.7°, θ_min_ = 2.6°
Absorption correction: multi-scan (*SADABS*; Bruker, 2009)	*h* = −9→9
*T*_min_ = 0.427, *T*_max_ = 0.921	*k* = −25→24
11951 measured reflections	*l* = −18→18

### Refinement

**Table:**

Refinement on *F*^2^	Primary atom site location: structure-invariant direct methods
Least-squares matrix: full	Secondary atom site location: difference Fourier map
*R*[*F*^2^ > 2σ(*F*^2^)] = 0.038	Hydrogen site location: inferred from neighbouring sites
*wR*(*F*^2^) = 0.089	H-atom parameters constrained
*S* = 1.01	*w* = 1/[σ^2^(*F*_o_^2^) + (0.034*P*)^2^ + 0.488*P*] where *P* = (*F*_o_^2^ + 2*F*_c_^2^)/3
4266 reflections	(Δ/σ)_max_ = 0.001
253 parameters	Δρ_max_ = 0.30 e Å^−^^3^
0 restraints	Δρ_min_ = −0.29 e Å^−^^3^

### Special details

**Table:**

Geometry. All esds (except the esd in the dihedral angle between two l.s. planes) are estimated using the full covariance matrix. The cell esds are taken into account individually in the estimation of esds in distances, angles and torsion angles; correlations between esds in cell parameters are only used when they are defined by crystal symmetry. An approximate (isotropic) treatment of cell esds is used for estimating esds involving l.s. planes.
Refinement. Refinement of F^2^ against ALL reflections. The weighted R-factor wR and goodness of fit S are based on F^2^, conventional R-factors R are based on F, with F set to zero for negative F^2^. The threshold expression of F^2^ > 2sigma(F^2^) is used only for calculating R-factors(gt) etc. and is not relevant to the choice of reflections for refinement. R-factors based on F^2^ are statistically about twice as large as those based on F, and R- factors based on ALL data will be even larger.

### Fractional atomic coordinates and isotropic or equivalent isotropic displacement parameters (Å^2^)

**Table:**

	*x*	*y*	*z*	*U*_iso_*/*U*_eq_	
Br1	0.24394 (5)	1.084300 (17)	0.06129 (3)	0.06481 (13)	
S1	0.21746 (11)	0.61887 (3)	0.38892 (5)	0.04655 (18)	
O1	0.2719 (3)	0.92795 (9)	0.43367 (12)	0.0453 (4)	
O2	0.2679 (3)	0.83501 (10)	0.51902 (14)	0.0567 (5)	
O3	0.2197 (3)	0.37198 (9)	0.04438 (13)	0.0518 (5)	
N1	0.2186 (3)	0.71059 (10)	0.25691 (15)	0.0403 (5)	
N2	0.2131 (3)	0.59752 (10)	0.19923 (16)	0.0459 (6)	
H1	0.2264	0.6102	0.1375	0.055*	
N3	0.2291 (3)	0.53132 (10)	0.23123 (16)	0.0397 (5)	
C1	0.2567 (4)	0.85797 (13)	0.43706 (19)	0.0402 (6)	
C2	0.2663 (4)	0.96192 (13)	0.34700 (19)	0.0407 (6)	
C3	0.2939 (4)	1.03179 (15)	0.3551 (2)	0.0515 (7)	
H3A	0.3160	1.0541	0.4171	0.062*	
C4	0.2880 (4)	1.06785 (14)	0.2703 (2)	0.0523 (7)	
H4A	0.3068	1.1150	0.2744	0.063*	
C5	0.2538 (4)	1.03353 (14)	0.1781 (2)	0.0446 (6)	
C6	0.2293 (4)	0.96446 (14)	0.1706 (2)	0.0439 (6)	
H6A	0.2081	0.9424	0.1085	0.053*	
C7	0.2358 (4)	0.92670 (12)	0.25618 (18)	0.0372 (6)	
C8	0.2190 (4)	0.85417 (13)	0.25783 (19)	0.0401 (6)	
H8A	0.1991	0.8297	0.1978	0.048*	
C9	0.2310 (4)	0.81934 (13)	0.34368 (18)	0.0372 (6)	
C10	0.2232 (4)	0.74489 (13)	0.34487 (18)	0.0366 (6)	
C11	0.2234 (4)	0.70361 (13)	0.42228 (19)	0.0430 (6)	
H11A	0.2264	0.7192	0.4854	0.052*	
C12	0.2177 (4)	0.64534 (12)	0.27085 (18)	0.0363 (6)	
C13	0.2343 (4)	0.48370 (12)	0.17014 (19)	0.0375 (6)	
H13A	0.2209	0.4934	0.1030	0.045*	
C14	0.2620 (4)	0.41341 (12)	0.20920 (18)	0.0348 (5)	
C15	0.3017 (4)	0.40043 (13)	0.3125 (2)	0.0445 (6)	
H15A	0.3043	0.4367	0.3559	0.053*	
C16	0.3374 (5)	0.33511 (14)	0.3522 (2)	0.0544 (8)	
H16A	0.3618	0.3275	0.4215	0.065*	
C17	0.3368 (5)	0.28129 (14)	0.2896 (2)	0.0557 (8)	
H17A	0.3650	0.2374	0.3170	0.067*	
C18	0.2947 (4)	0.29182 (13)	0.1862 (2)	0.0479 (7)	
H18A	0.2917	0.2550	0.1437	0.057*	
C19	0.2572 (4)	0.35707 (13)	0.14584 (18)	0.0376 (6)	
C20	0.2017 (5)	0.31519 (14)	−0.0228 (2)	0.0530 (7)	
H20A	0.1687	0.3315	−0.0920	0.080*	
H20B	0.0979	0.2852	−0.0220	0.080*	
H20C	0.3262	0.2908	0.0005	0.080*	

### Atomic displacement parameters (Å^2^)

**Table:**

	*U*^11^	*U*^22^	*U*^33^	*U*^12^	*U*^13^	*U*^23^
Br1	0.0782 (2)	0.0614 (2)	0.0665 (2)	−0.00399 (17)	0.04110 (19)	0.01182 (16)
S1	0.0669 (5)	0.0376 (4)	0.0421 (4)	−0.0001 (3)	0.0291 (3)	0.0017 (3)
O1	0.0660 (12)	0.0354 (10)	0.0349 (10)	0.0021 (9)	0.0203 (9)	−0.0065 (8)
O2	0.0930 (16)	0.0460 (11)	0.0367 (11)	0.0041 (10)	0.0315 (11)	−0.0039 (9)
O3	0.0867 (14)	0.0353 (10)	0.0391 (10)	−0.0008 (10)	0.0310 (10)	−0.0042 (8)
N1	0.0546 (13)	0.0317 (12)	0.0366 (12)	−0.0013 (10)	0.0202 (10)	−0.0057 (9)
N2	0.0760 (16)	0.0297 (12)	0.0394 (12)	−0.0014 (11)	0.0307 (12)	−0.0028 (9)
N3	0.0529 (13)	0.0285 (11)	0.0404 (12)	−0.0002 (10)	0.0213 (10)	−0.0002 (9)
C1	0.0459 (15)	0.0371 (15)	0.0377 (14)	0.0032 (12)	0.0167 (12)	−0.0057 (12)
C2	0.0451 (15)	0.0357 (15)	0.0408 (15)	0.0018 (12)	0.0166 (12)	−0.0033 (12)
C3	0.0630 (19)	0.0439 (17)	0.0441 (16)	−0.0017 (14)	0.0176 (14)	−0.0127 (13)
C4	0.0640 (19)	0.0327 (15)	0.0591 (18)	−0.0057 (14)	0.0234 (16)	−0.0050 (13)
C5	0.0454 (16)	0.0408 (16)	0.0516 (16)	0.0001 (13)	0.0235 (13)	0.0055 (13)
C6	0.0511 (16)	0.0444 (16)	0.0398 (15)	−0.0008 (13)	0.0219 (13)	−0.0045 (12)
C7	0.0410 (14)	0.0340 (14)	0.0366 (14)	0.0012 (11)	0.0155 (12)	−0.0064 (11)
C8	0.0481 (16)	0.0386 (15)	0.0349 (14)	−0.0009 (12)	0.0179 (12)	−0.0093 (11)
C9	0.0400 (14)	0.0367 (14)	0.0359 (14)	0.0016 (11)	0.0159 (12)	−0.0077 (11)
C10	0.0391 (14)	0.0369 (14)	0.0336 (13)	0.0012 (11)	0.0141 (11)	−0.0066 (11)
C11	0.0578 (17)	0.0398 (15)	0.0356 (14)	0.0011 (13)	0.0231 (13)	−0.0046 (11)
C12	0.0444 (15)	0.0334 (14)	0.0324 (13)	−0.0006 (11)	0.0165 (12)	−0.0026 (11)
C13	0.0485 (15)	0.0327 (14)	0.0336 (13)	−0.0012 (12)	0.0187 (12)	−0.0011 (11)
C14	0.0400 (13)	0.0296 (13)	0.0376 (14)	−0.0030 (11)	0.0184 (11)	−0.0013 (11)
C15	0.0605 (18)	0.0359 (15)	0.0404 (15)	−0.0032 (13)	0.0236 (14)	−0.0031 (12)
C16	0.082 (2)	0.0437 (17)	0.0405 (16)	0.0001 (15)	0.0280 (16)	0.0043 (13)
C17	0.079 (2)	0.0344 (15)	0.0556 (18)	0.0057 (15)	0.0293 (17)	0.0109 (13)
C18	0.0691 (19)	0.0311 (15)	0.0502 (16)	−0.0027 (13)	0.0309 (15)	−0.0047 (12)
C19	0.0454 (15)	0.0337 (14)	0.0380 (14)	−0.0032 (11)	0.0211 (12)	−0.0013 (11)
C20	0.073 (2)	0.0473 (17)	0.0419 (16)	−0.0035 (15)	0.0259 (15)	−0.0133 (13)

### Geometric parameters (Å, °)

**Table:**

Br1—C5	1.896 (3)	C6—H6A	0.9300
S1—C11	1.718 (3)	C7—C8	1.424 (3)
S1—C12	1.740 (2)	C8—C9	1.359 (3)
O1—C2	1.375 (3)	C8—H8A	0.9300
O1—C1	1.375 (3)	C9—C10	1.457 (3)
O2—C1	1.209 (3)	C10—C11	1.355 (3)
O3—C19	1.374 (3)	C11—H11A	0.9300
O3—C20	1.430 (3)	C13—C14	1.464 (3)
N1—C12	1.291 (3)	C13—H13A	0.9300
N1—C10	1.396 (3)	C14—C15	1.387 (3)
N2—N3	1.360 (3)	C14—C19	1.409 (3)
N2—C12	1.365 (3)	C15—C16	1.377 (4)
N2—H1	0.9449	C15—H15A	0.9300
N3—C13	1.277 (3)	C16—C17	1.371 (4)
C1—C9	1.461 (3)	C16—H16A	0.9300
C2—C3	1.379 (4)	C17—C18	1.377 (4)
C2—C7	1.389 (3)	C17—H17A	0.9300
C3—C4	1.371 (4)	C18—C19	1.379 (3)
C3—H3A	0.9300	C18—H18A	0.9300
C4—C5	1.392 (4)	C20—H20A	0.9600
C4—H4A	0.9300	C20—H20B	0.9600
C5—C6	1.361 (4)	C20—H20C	0.9600
C6—C7	1.397 (3)		
			
C11—S1—C12	87.96 (12)	C11—C10—C9	128.1 (2)
C2—O1—C1	122.48 (18)	N1—C10—C9	117.2 (2)
C19—O3—C20	116.75 (19)	C10—C11—S1	111.30 (18)
C12—N1—C10	109.9 (2)	C10—C11—H11A	124.4
N3—N2—C12	115.7 (2)	S1—C11—H11A	124.4
N3—N2—H1	121.9	N1—C12—N2	124.4 (2)
C12—N2—H1	121.1	N1—C12—S1	116.16 (17)
C13—N3—N2	119.5 (2)	N2—C12—S1	119.44 (18)
O2—C1—O1	115.2 (2)	N3—C13—C14	117.9 (2)
O2—C1—C9	126.9 (2)	N3—C13—H13A	121.0
O1—C1—C9	117.8 (2)	C14—C13—H13A	121.0
O1—C2—C3	116.9 (2)	C15—C14—C19	117.5 (2)
O1—C2—C7	120.9 (2)	C15—C14—C13	120.3 (2)
C3—C2—C7	122.1 (2)	C19—C14—C13	122.1 (2)
C4—C3—C2	118.9 (2)	C16—C15—C14	121.5 (2)
C4—C3—H3A	120.5	C16—C15—H15A	119.3
C2—C3—H3A	120.5	C14—C15—H15A	119.3
C3—C4—C5	119.6 (3)	C17—C16—C15	119.9 (3)
C3—C4—H4A	120.2	C17—C16—H16A	120.0
C5—C4—H4A	120.2	C15—C16—H16A	120.0
C6—C5—C4	121.4 (2)	C16—C17—C18	120.4 (3)
C6—C5—Br1	119.5 (2)	C16—C17—H17A	119.8
C4—C5—Br1	119.1 (2)	C18—C17—H17A	119.8
C5—C6—C7	119.9 (2)	C17—C18—C19	119.8 (2)
C5—C6—H6A	120.0	C17—C18—H18A	120.1
C7—C6—H6A	120.0	C19—C18—H18A	120.1
C2—C7—C6	118.0 (2)	O3—C19—C18	123.3 (2)
C2—C7—C8	117.5 (2)	O3—C19—C14	115.9 (2)
C6—C7—C8	124.5 (2)	C18—C19—C14	120.8 (2)
C9—C8—C7	122.5 (2)	O3—C20—H20A	109.5
C9—C8—H8A	118.7	O3—C20—H20B	109.5
C7—C8—H8A	118.7	H20A—C20—H20B	109.5
C8—C9—C10	121.4 (2)	O3—C20—H20C	109.5
C8—C9—C1	118.7 (2)	H20A—C20—H20C	109.5
C10—C9—C1	119.9 (2)	H20B—C20—H20C	109.5
C11—C10—N1	114.7 (2)		
			
C12—N2—N3—C13	−177.2 (2)	C1—C9—C10—C11	4.9 (4)
C2—O1—C1—O2	178.6 (2)	C8—C9—C10—N1	4.9 (4)
C2—O1—C1—C9	−0.9 (3)	C1—C9—C10—N1	−173.7 (2)
C1—O1—C2—C3	−176.6 (2)	N1—C10—C11—S1	−0.1 (3)
C1—O1—C2—C7	2.9 (4)	C9—C10—C11—S1	−178.8 (2)
O1—C2—C3—C4	−179.5 (3)	C12—S1—C11—C10	0.6 (2)
C7—C2—C3—C4	1.0 (4)	C10—N1—C12—N2	179.9 (2)
C2—C3—C4—C5	0.3 (4)	C10—N1—C12—S1	1.2 (3)
C3—C4—C5—C6	−1.1 (4)	N3—N2—C12—N1	174.9 (2)
C3—C4—C5—Br1	179.5 (2)	N3—N2—C12—S1	−6.4 (3)
C4—C5—C6—C7	0.8 (4)	C11—S1—C12—N1	−1.1 (2)
Br1—C5—C6—C7	−179.84 (19)	C11—S1—C12—N2	−179.8 (2)
O1—C2—C7—C6	179.2 (2)	N2—N3—C13—C14	176.9 (2)
C3—C2—C7—C6	−1.3 (4)	N3—C13—C14—C15	−6.5 (4)
O1—C2—C7—C8	−2.6 (4)	N3—C13—C14—C19	176.0 (2)
C3—C2—C7—C8	176.9 (2)	C19—C14—C15—C16	0.7 (4)
C5—C6—C7—C2	0.4 (4)	C13—C14—C15—C16	−177.0 (3)
C5—C6—C7—C8	−177.7 (2)	C14—C15—C16—C17	0.9 (5)
C2—C7—C8—C9	0.3 (4)	C15—C16—C17—C18	−2.0 (5)
C6—C7—C8—C9	178.4 (3)	C16—C17—C18—C19	1.4 (5)
C7—C8—C9—C10	−176.9 (2)	C20—O3—C19—C18	5.8 (4)
C7—C8—C9—C1	1.7 (4)	C20—O3—C19—C14	−176.2 (2)
O2—C1—C9—C8	179.2 (3)	C17—C18—C19—O3	178.1 (3)
O1—C1—C9—C8	−1.4 (4)	C17—C18—C19—C14	0.2 (4)
O2—C1—C9—C10	−2.1 (4)	C15—C14—C19—O3	−179.3 (2)
O1—C1—C9—C10	177.3 (2)	C13—C14—C19—O3	−1.7 (4)
C12—N1—C10—C11	−0.7 (3)	C15—C14—C19—C18	−1.3 (4)
C12—N1—C10—C9	178.2 (2)	C13—C14—C19—C18	176.4 (2)
C8—C9—C10—C11	−176.5 (3)		

### Hydrogen-bond geometry (Å, °)

**Table:**

*D*—H···*A*	*D*—H	H···*A*	*D*···*A*	*D*—H···*A*
N2—H1···O2^i^	0.94	2.10	3.021 (3)	164

Symmetry codes: (i) *x*, −*y*+3/2, *z*−1/2.
